# Chronic intermittent hypoxia due to obstructive sleep apnea slightly alters nutritional status: a pre-clinical study

**DOI:** 10.3389/fnut.2023.1250529

**Published:** 2023-10-30

**Authors:** Charlotte Breuillard, Sophie Moulin, Sophie Bouyon, Morgane Couchet, Christophe Moinard, Elise Belaidi

**Affiliations:** ^1^Université Grenoble Alpes, Inserm, Laboratory of Fundamental and Applied Bioenergetics (LBFA), Grenoble, France; ^2^Université Grenoble Alpes, Inserm, Laboratory HP2, Grenoble, France

**Keywords:** chronic intermittent hypoxia, obstructive sleep apnea, nutritional status, anorexia, protein metabolism

## Abstract

Obstructive sleep apnea syndrome (OSAS) is associated with chronic intermittent hypoxia (cIH) that causes disturbances in glucose and lipid metabolism. Animals exposed to cIH show lower body weight and food intake, but the protein-energy metabolism has never been investigated. Here, to address the gap, we studied the impact of cIH on nutritional status in rats. A total of 24 male Wistar rats were randomized into 3 groups (*n* = 8): a control group (Ctrl), a cIH group (cIH) exposed to cIH (30 s 21–30 s 5% fraction of inspired oxygen, 8 h per day, for 14 days), and a pair-fed group (PF) exposed to normoxia with food intake adjusted to the intake of the cIH group rats with anorexia. Body weight and food intake were measured throughout the study. After 14 days, the rats were euthanized, the organs were collected, weighed, and the liver, intestine mucosa, and muscles were snap-frozen to measure total protein content. Food intake was decreased in the cIH group. Body weight was significantly lower in the cIH group only (−11%, *p* < 0.05). Thymus and liver weight as well as *EDL* protein content tended to be lower in the cIH group than in the Ctrl and PF groups. Jejunum and ileum mucosa protein contents were lower in the cIH group compared to the PF group. cIH causes a slight impairment of nutritional status and immunity. This pre-clinical work argues for greater consideration of malnutrition in care for OSAS patients. Further studies are warranted to devise an adequate nutritional strategy.

## Introduction

Obstructive sleep apnea syndrome (OSAS) is characterized by recurrent episodes of collapse of the upper airway during sleep resulting in chronic intermittent hypoxia (cIH). OSAS induces disturbances in glucose and lipid metabolism (1, 2). As OSAS is strongly associated with obesity (1), the issue of whether OSAS alters protein-energy metabolism has never been explored, despite the growing body of evidence that sarcopenic obesity negatively affects patient outcomes, increasing the risks for frailty, disability, and morbimortality (3–5).

A preclinical model demonstrated that cIH, which is the major feature of OSAS, is a critical component of glucose homeostasis disturbances, including insulin resistance characterized by decreased insulin sensitivity and increased HOMA-IR (homeostasis model assessment of insulin resistance), and dyslipidemia characterized by increased total cholesterol, LDL (low density lipoprotein), or triglyceride levels (6–11). However, animals exposed to cIH showed decreased food intake, especially along hypoxia exposure, that was associated with a lower body weight (7, 8, 12), which suggests an alteration of nutritional status.

To the best of our knowledge, altered protein-energy metabolism under cIH has never been studied. Here, to address this gap, this study set out to investigate the impact of cIH on nutritional status in rats.

## Materials and methods

### Experimental design

#### Animals

All the procedures were registered as compliant with European Directives on the care and use of animals for research purposes, and approved by the French Ministry of Research (APAFIS #201603301129626-V3).

The study used 24 male Wistar rats aged 6–7 weeks old. The rats were housed 6 per cage in a temperature-controlled facility (22 ± 2°C) on a 12 h light/dark cycle for an 8-day acclimatization period. Water and food (16.9% proteins, 4.3% lipids; LASQCDiet R16-R) were provided *ad libitum*.

The rats were then randomized into 3 groups (*n* = 8 per group): a healthy control group (Ctrl) exposed to normoxia and fed *ad libitum*; a cIH group (cIH) exposed to cIH (see below) and fed *ad libitum*; a pair-fed group (PF) exposed to normoxia with food intake adjusted to intake of the cIH group who suffered of anorexia to rule out the effect of decreased food intake decrease observed in cIH.

During the study, rats were separated into individual cages in order to measure their food intake (the difference between the food given the day before and the food still in the cage) (13, 14). Body weight and food intake were monitored throughout the study to evaluate nutritional status (15).

#### Intermittent hypoxia

Briefly, rats were exposed to cIH (cIH; 30 s-O_2_ 21%/30 s-O_2_ 5%; 8 h.d^–1^) in their cages for 14 days or to normoxia (Ctrl and PF; 30 s-O_2_ 21%/30 s-O_2_ 21%; 8 h.d^–1^) to reproduce equivalent levels of noise and air turbulence related to gas circulation. Fraction of inspired oxygen (FIO_2_) was monitored with a ML206-model gas analyzer (ADInstruments, Dunedin, New Zealand) (7, 8).

### Euthanasia and sample collection

At D14, the rats were fasted for 3 h -then euthanized by exsanguination. Blood was collected into capillary tubes for hematocrit determination.

The heart, liver, kidney, spleen, thymus, the *extensor digitorum longus* (*EDL*), *tibialis* and *soleus* muscles (right and left), and the jejunum and ileum (proximal part) mucosa were weighed. The muscles, liver and intestinal mucosa were snap-frozen in liquid nitrogen and stored at −80°C until analysis.

### Protein content analysis

Frozen *EDL*, *tibialis*, *soleus*, liver, jejunum mucosa and ileum mucosa were ground and homogenized in 10 volumes of ice-cold 10% trichloroacetic acid, 0.5 mmol/l EDTA. After delipidation with ethanol/ether (1:1 vol/vol), the pellets were dissolved in NaOH 1N (4 mL/100 mg tissue, 12 h at +40°C). Then, total protein content was determined by a method based on bicinchoninic acid (Pierce™ BCA Protein Assay Kit; ThermoScientific, Rockford, IL, USA) (13, 16).

### Statistical analysis

Results are reported as means ± SEM. After checking the data for normality, food intake and body weight change were studied using two-way (group × time) repeated-measures analysis of variance (ANOVA). For the other parameters, if D’Agostino-Pearson’s K^2^ test for normality passed (*p* > 0.05), we used ANOVA followed by Tukey’s *post-hoc* test, and if the normality test failed, we used a Kruskal–Wallis one-way analysis followed by Dunn’s *post-hoc* test. Differences were considered statistically significant at *p* < 0.05.

## Results

### Hematocrit

**FIGURE 1:**
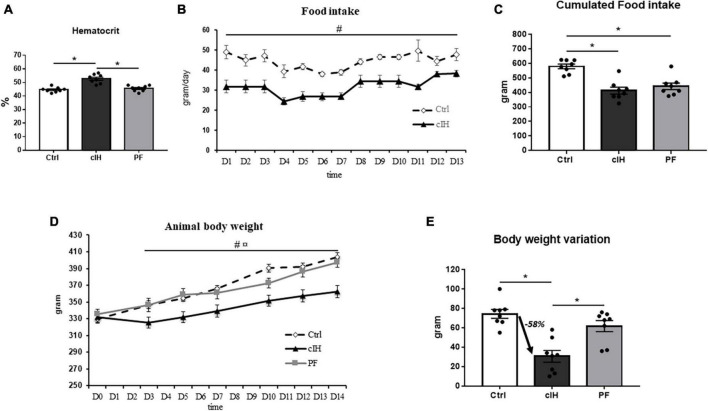
Model validation and rat food intake and body weight variation. **(A)** Hematocrit, **(B)** rat food intake, **(C)** 14-day cumulative food intake, **(D)** rat body weight, **(E)** rat body weight variation between D0 and D14. Healthy control rats (Ctrl; *n* = 8) were under normoxia and fed *ad libitum*. The chronic intermittent hypoxia group (cIH; *n* = 8) was exposed to intermittent hypoxia [60-s intermittent hypoxia cycles: 30 s at 5% fraction of inspired oxygen (FIO_2_) and 30 s at 21% FIO_2_, for 8 h a day] and fed *ad libitum*. The pair-fed group (PF; *n* = 8) was under normoxia and had the same food consumption as the cIH-group rats that showed anorexia. The rats were euthanized at D14. Results are expressed as means ± SEM. *p < 0.05; # cIH vs. Ctrl, *p* < 0.05; 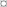 cIH vs. PF, *p* < 0.05.

Hematocrit was higher in the cIH group compared to the other groups (Figure 1A), thus confirming the exposure to hypoxia.

### Food intake and body weight

Food intake was lower in cIH rats than in Ctrl rats throughout the exposure (Figure 1B). A total of 30% lower cumulative food intake was observed after 14 days of cIH (Figure 1C).

Body weight was lower in cIH rats than in Ctrl and PF rats throughout the exposure (Figure 1D), with a 58% lower total body weight gain after 14 days of cIH (Figure 1E).

### Organ weights

**FIGURE 2:**
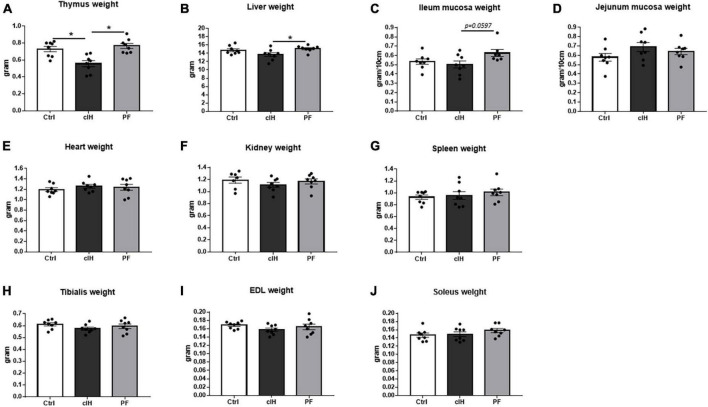
Organ weights. Weights of **(A)** thymus, **(B)** liver, **(C)** ileum mucosa, **(D)** jejunum mucosa, **(E)** heart, **(F)** kidney, **(G)** spleen, **(H)** *tibialis* muscle, **(I)** *EDL* muscle, and **(J)** *soleus* muscle. Healthy control rats (Ctrl; *n* = 8) were under normoxia and fed *ad libitum*. The chronic intermittent hypoxia group (cIH; *n* = 8) was exposed to intermittent hypoxia [60-s intermittent hypoxia cycles: 30 s at 5% fraction of inspired oxygen (FIO_2_) and 30 s at 21% FIO_2_, for 8 h a day] and fed *ad libitum*. The pair-fed group (PF; *n* = 8) was under normoxia and had the same food consumption as the cIH-group rats that showed anorexia. The rats were euthanized at D14. Results are expressed as means ± SEM. *p < 0.05.

Thymus weight was lower in cIH rats compared to Ctrl and PF rats (Figure 2A). Liver weight was lower and ileum mucosa weight tended to be lower (*p* = 0.0597) in the cIH group compared to the PF group (Figures 2B, C). Heart, kidney, spleen, jejunum mucosa and muscle weights were not modified by cIH or anorexia (Figures 2D–J).

### Tissue protein contents

**FIGURE 3:**
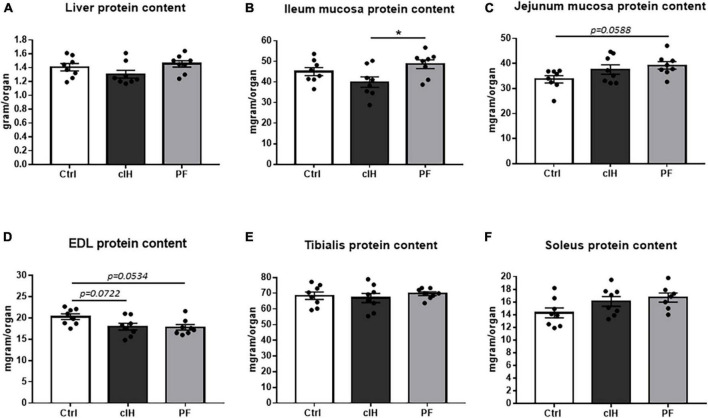
Tissue protein content. Protein content of **(A)** liver, **(B)** ileum mucosa, **(C)** jejunum mucosa, **(D)** *EDL* muscle, **(E)** *tibialis* muscle, and **(F)** *soleus* muscle. Healthy control rats (Ctrl; *n* = 8) were under normoxia and fed *ad libitum*. The chronic intermittent hypoxia group (cIH; *n* = 8) was exposed to intermittent hypoxia [60-s intermittent hypoxia cycles: 30 s at 5% fraction of inspired oxygen (FIO_2_) and 30 s at 21% FIO_2_, for 8 h a day] and fed *ad libitum*. The pair-fed group (PF; *n* = 8) was under normoxia and had the same food consumption as the cIH-group rats that showed anorexia. The rats were euthanized at D14. Results are expressed as means ± SEM. *p < 0.05.

Liver protein content showed no difference between the 3 groups (Figure 3A). Ileum mucosa protein content was lower in cIH rats compared to PF rats, whereas jejunum mucosa protein content tended to be higher (*p* = 0.0588) in PF rats compared to Ctrl-group rats (Figures 3B, C). In muscle, *EDL* protein content tended to be lower in cIH and PF rats than in Ctrl rats (*p* = 0.0534 and *p* = 0.0722, respectively) whereas *tibialis* and *soleus* protein content showed no difference between the 3 groups (Figures 3D–F).

## Discussion

OSAS is found mainly in obese patients. For this reason, no study yet has tested whether OSAS-induced cIH affects nutritional status, whereas malnutrition during obesity (i.e., sarcopenic obesity) is emerging as a major problem (3–5). First, as previously described, we validated our model by demonstrating that cIH induced an increase in hematocrit (12). The hypoxia inducible factor-1 (HIF-1)-related erythropoietin (EPO) gene transcription is an adaptative answer to the decrease in PO_2_ and is responsible for an increase in hematocrit (17). In the context of cIH, HIF-1 is strongly activated (18); thus its activation on EPO gene is one of the main explanation to observe an increase in hematocrit induced by cIH. Then, here, for the first time, we evaluated the effect of cIH, the critical component in terms of metabolic effects of OSAS, on nutritional status in rats. We showed that cIH slightly negatively impacts nutritional status, independently of the associated anorexia, causing decreased body weight and a modest alteration of protein metabolism in intestine and muscle.

cIH had modest effects on nutritional status but with signs of altered protein metabolism. As expected, we observed anorexia, as reported in animal models of cIH (7, 8, 12). In the same way, previous studies reported an impact of cIH on feeding behavior that is tightly controlled by the central nervous system. The increase in plasma leptin level has been proposed to explain an anorexigen effect (7). However, in this study we suggest that cIH alters some specific O_2_-sensitive pathways controlling feeding behavior. Among them, adiponectin, an adipokine recognized to counteract the effect of leptin, is decreased under IH and in sleep apnea patients (19). Anorexia is usually observed in stress situations (e.g., sepsis, cancer-related chemotherapy) (13, 14, 20), but most of these stresses have been applied acutely and are associated with short-term anorexia followed by a rapid increase in food intake to redress the loss of energy intake. Here, the anorexia persisted throughout the 14 days of exposure. This suggests that long-term anorexia (even if quite moderate, i.e., 35%) in cIH could lead to malnutrition and thus all the consequences of malnutrition such as sarcopenia and dysimmunity, as well as decreased quality of life and increased morbimortality (3–5). Interestingly, the loss of body weight was not related to anorexia (no effect of pair feeding) but was specifically related to cIH. One explanation for this cIH-related body weight loss could be the metabolic disturbances induced by cIH, particularly in adipose tissue, such as morphological changes in adipose tissue including altered inflammatory profiles, activation of HIF-1, and sympathetic activation-induced lipolysis (21).

As body weight loss is usually associated with sarcopenia and dysimmunity, we explored protein metabolism and observed some alterations caused by cIH itself. First, cIH clearly induced disturbances in protein metabolism, especially in the intestinal tract, with no intestinal mucosa response to anorexia. This is consistent with previous works showing that hypoxia leads to gastrointestinal disorders, due in part to disrupted intestinal microbiota (22), and that OSAS is characterized by microbiota disruption and impaired intestinal barrier function (23), but there has been no previous work on intestinal protein metabolism under cIH.

Second, cIH induced an alteration in the liver response to anorexia. Substantial alterations in hepatic glucose metabolism (i.e., systemic insulin resistance) have already been observed in our rodent cIH model (7, 8), but here we found no change in hepatic protein content. However, since the canonical response to insulin (i.e., ^308Thr/473Ser–P^PKB/PKB) has been demonstrated to be altered in liver, adipose tissue and striated skeletal muscle (7), we have to consider a direct impact of canonical insulin signaling pathway impairment on protein synthesis. In line with systemic metabolic disturbances, mitochondrial function and structure alterations could also explain an alteration in protein metabolism since adenosine triphosphate (ATP) production is essential for protein synthesis. Besides cIH induces mitochondrial dysfunction and structural damages in several organs such as striated skeletal muscle (24), liver (25), heart (26) and brain (27). Finally, since a decade, cIH has also been demonstrated to induce endoplasmic reticulum (ER) stress in several organs such as liver (28), heart (29) and brain (30). Since ER is involved in protein folding, we cannot exclude the impact of cIH on misfolded protein clearance and metabolism alteration.

Third, as previously observed by Iiyori et al. (31), we expected to see an alteration of protein metabolism mainly in oxidative muscle (i.e., *soleus*) in response to the lack of oxygen. Instead, we observed that protein metabolism tended to be altered in glycolytic muscle (i.e., *EDL*), and in both anorexic groups, suggesting that this response is not specifically related to cIH. The lack of effects of cIH on muscle protein metabolism observed in our study could be explained by metabolic adaptations of the muscle [for review see (32)]. Hence, previous data on hypoxia showed that muscle protein synthesis was maintained in myotubes whereas oxygen consumption was declined (33), and was even increased in rat submitted to hypoxia (34). As protein synthesis is an important driver of energy expenditure (i.e., accounting for approximately 20% of energy consumption in cells) (35), the increase of muscle protein synthesis should be supported by a modulation of energy metabolism. Therefore, the increase of muscle protein synthesis could be associated with an increase of the energy expenditure. Such results suggest that even if muscle weight and muscle protein content are not modified, an increase in muscle protein turnover, raising energy expenditure and leading to body weight loss, cannot be excluded. Further studies exploring deeply the effect of cIH on body composition and energy expenditure would be of major interest.

According to the literature, metabolic modifications (i.e., body weight and lipid metabolism), like the cardiovascular alterations associated with cIH, are highly dependent on the severity of cIH (5 vs. 10% FIO_2_) and the cIH pattern (number of hours/day and FIO_2_ percentage) (9, 31, 36). For example, body weight was not modified in rats under slight cIH (10%) but was decreased under severe cIH (5% FIO_2_) (9, 37). Here, under severe cIH (5% FIO_2_), we observed the same body weight loss (i.e., 10%) but only slight alterations in protein metabolism, suggesting that protein metabolism alterations may not be dependent on the severity and pattern of cIH. Further investigations are needed to better understand the impact of cIH on protein metabolism.

The consequences of hypoxia on immune cells are well known from decades [For recent review see (38)], but the knowledge on the effects of cIH are much more scattered. In our study, cIH caused thymic involution, which reflects immunosuppression and malnutrition (39). Immune dysfunction, at cellular level, has been found in OSAS, but it is difficult to separate the cause between cIH and OSA-related comorbidities (40, 41). In our work, we confirm a direct effect of cIH on immune status. Among the explanations which could be proposed, one is a crosstalk between IH and innate immunity previously revealed to involve the activation of HIF-1 and NF-kB (42). Although further experiments are needed to more deeply explore immune function, this study nevertheless showed that cIH has a share of responsibility in immune dysfunction, which is not surprising given that immune cell function requires oxygen.

## Conclusion

This preliminary study suggests that cIH itself can affect both nutritional and immune status. This pre-clinical work argues strongly for greater consideration of malnutrition in care for OSAS patients, and further research is warranted in order to devise an adequate nutritional strategy for OSAS patients.

## Data availability statement

The original contributions presented in this study are included in this article/supplementary material, further inquiries can be directed to the corresponding author.

## Ethics statement

The animal study was approved by the French Ministry of Research (APAFIS #201603301129626-V3). The study was conducted in accordance with the local legislation and institutional requirements.

## Author contributions

CB, SM, CM, and EB contributed to conception and design of the study. CB, SM, SB, MC, and EB performed the study. CB analyzed the results, performed the statistical analysis, and wrote the first draft of the manuscript. All authors contributed to manuscript revision, read, and approved the submitted version.
